# Locus coeruleus lesions and PCOS: role of the central and peripheral sympathetic nervous system in the ovarian function of rat

**Published:** 2012-03

**Authors:** Farideh Zafari Zangeneh, Alireza Abdollahi, Fatemeh Aminee, Mohammad Mahdi Naghizadeh

**Affiliations:** 1 *Vali-e-Asr Reproductive Health Research Center, Tehran University of Medical Sciences, Tehran, Iran.*; 2 *Vali-e-Asr Hospital, Pathology Laboratory, Tehran University of Medical Sciences, Tehran, Iran.*; 3 *Veterinary Faculty, Tehran University, Tehran, Iran.*; 4 *Central Biostatistics, Fasa University of Medical Sciences, Fasa, Iran. *

**Keywords:** *Polycystic ovary syndrome*, *Locus coeruleus*, *Gonadotropines*, *Estradiol*, *Ovary morphology*, *Rat*

## Abstract

**Background: “**Polycystic ovary syndrome (PCOS) is a complex endocrine and metabolic disorder associated with ovulatory dysfunction”. “Autonomic and central nervous systems play important roles in the regulation of ovarian physiology”. The noradrenergic nucleus locus coeruleus (LC) plays a central role in the regulation of the sympathetic nervous system and synaptically connected to the preganglionic cell bodies of the ovarian sympathetic pathway and its activation is essential to trigger spontaneous or induced LH surges. This study evaluates sympathetic outflow in central and peripheral pathways in PCO rats.

**Objective:** Our objectives in this study were (1) to estimate LC activity in rats with estradiol valerate (EV)-induced PCO; (2) to antagonized alpha2a adrenoceptor in systemic conditions with yohimbine.

**Materials and Methods:** Forty two rats were divided into two groups: 1) LC and yohimbine and 2) control. Every group subdivided in two groups: eighteen rats were treated with estradiol valerate for induction of follicular cysts and the remainders were sesame oil groups.

**Results:** Estradiol concentration was significantly augmented by the LC lesion in PCO rats (p<0.001), while LC lesion could not alter serum concentrations of LH and FSH, like yohimbine. The morphological observations of ovaries of LC lesion rats showed follicles with hyperthecosis, but yohimbine reduced the number of cysts, increased corpus lutea and developed follicles.

**Conclusion:** Rats with EV-induced PCO increased sympathetic activity. LC lesion and yohimbine decreased the number of cysts and yohimbine increased corpus lutea and developed follicles in PCO rats.

## Introduction

It is estimated that 6-7% of women of reproductive age have polycystic ovarian syndrome (PCOS) (1). Polycystic ovary syndrome (PCOS), the most common female endocrine disorder, is a complex and heterogenic disease with unknown etiology. PCOS is characterized by reproductive disturbances including chronic anovulation, hyper-androgenism, and polycystic ovaries (2). Autonomic and central nervous systems play important roles in the regulation of ovarian physiology (3). 

“It is well known that one of the major neurotransmitters that control LH secretion is norepinephrine (NE)”. The NE turnover in the preoptic area parallels changes in circulating LH concentrations (4). Infusion of noradrenaline intracerebroventricularly (5) in the rat, suppresses LH pulses, as does electrical stimulation of the brain stem ascending noradrenergic pathways (6), “thus suggesting that increases in noradrenergic activity results in a suppression of the GnRH pulse generator”. In contrast, peripheral administration of -adrenergic receptor antagonists decreases the frequency of the GnRH pulse generator (7).

The finding that both reduction and increase in adrenergic receptor activity have the same effect on pulsatile LH secretion led Leng and colleagues (8) “to propose that fluctuating patterns of adrenergic receptor activity are essential for pulsatile GnRH release, a postulate substantiated by theoretical modeling experiments”. The syndrome is associated with peripheral and central factors that influence sympathetic nerve activity. Thus, the sympathetic nervous system may be an important factor in the development and maintenance of PCOS (9). 

“Although the involvement of the sympathetic nervous system has been suggested in PCOS, this is the first time that direct intraneural recordings of sympathetic nerve activity have been obtained in women with PCOS”. PCOS is associated with hyperandrogenemia, hyperinsulinemia (10), and insulin resistance, as well as abdominal obesity, cardiovascular disease and obstructive sleep apnea (11), all factors hypothesized to be associated with increased activity of the sympathetic nervous system (10). 

Increasing evidence also supports the presence of a primary defect in ovarian and adrenal steroidogenesis in PCOS (12), resulting in elevated androgen production, both basally and in response to LH (13). Women with PCOS have significantly higher sympathetic nerve activity than their matched controls and the increased sympathetic outflow is related to hormonal and metabolic features (14). 

“The locus coeruleus (LC), a well-delineated cluster of noradrenaline-containing neurones located adjacent to the fourth ventricle in the pontine brainstem is the major noradrenergic nucleus in the brain. The LC activity is remarkably synchronized, producing a coordinated release of noradrenaline (NA) throughout the central nervous system.” 

It is estimated that ~50% of all of the noradrenergic projections in the central nervous system originate in the LC (15) and several brainstem catecholaminergic neurons are known to innervate the sympathetic preganglionic neurons and thereby are potential modulators of the sympathetic nervous system response to stress (16, 17). For example, chronic stress promotes PCO in rats, which seems to be initiated by an increased central noradrenergic tonus dictated by an augmentation in the LC activity (18). 

Electrolytic lesions of the LC block the preovulatory surge of LH. This blockade of LH surge is accompanied by a decrease in the NE content in the medial preoptic area (MPOA) and medial basal hypothalamus (MBH) (19). These findings strongly suggest that NE from the LC plays an important role mediating the positive feedback action of E_2_ on LH secretion (20). 

Experimental induction of a polycystic ovarian syndrome (PCOS) in rodents by the administration of a single dose of estradiol valerate (EV) results in activation of the peripheral sympathetic neurons that innervate the ovary. This activation is evidenced by an increased capacity of ovarian nerve terminals to incorporate and release norepinephrine (NE), an increase in ovarian NE content, and a decrease in ovarian beta-adrenergic receptor number in the ovarian compartments receiving catecholaminergic innervation. 

This increased ovarian sympathetic outflow suggested by these alterations in catecholamine homeostasis was accompanied by a thecal cell-interstitial tissue selective down-regulation of beta-adrenergic receptors; the beta-adrenergic receptor concentration in these sympathetically innervated ovarian compartments was significantly lower in PCO than during the estrous phase of the estrous cycle, a time at which the beta-adrenergic receptor concentration reaches its lowest levels in normal cycling ovaries (21). 

The sympathic ovary nerve (SON) transection also reduced the elevated levels of ovarian NE resulting from EV treatment and caused up-regulation of beta-adrenoreceptors. Most importantly, SON transection restored estrous cyclicity and ovulatory capacity. The results indicate that the increased output of ovarian steroids in PCOS is at least in part due to an enhanced responsiveness of the gland to both catecholaminergic and gonadotropin stimulation.

 The ability of SON transection to restore a normal response indicates that the alteration in steroid output results from a deranged activation of selective components of the noradrenergic innervation to the ovary. These findings support the concept that an alteration in the neurogenic control of the ovary contributes to the etiology of PCOS (22). 

The aim of this study was to evaluate the effects of LC (central) lesion and yohimbine (peripheral) in modeling of PCOS in rat, which occurs during estrus phase.

## Materials and methods

**Animals and care**

“Adult female Wistar rats weighing 220-230 g (7–8 wk of age) from the animal house of the Pastor Institute were kept in a central animal care facility under a 12-h light, 12-h dark cycle and controlled temperature (24±0.5^o^C). Food and water were provided ad libitum”. 

Vaginal smears were taken daily and only rats showing estrous phase were used in the experiment. Study groups (forty two rats) were divided into two groups: 1) LC and yohimbine, 2) control (sesame oil) and intact group. Every group subdivided in two groups: eighteen rats were treated with estradiol valerate for induction of follicular cysts (PCO modeling) and the remainders were sesame oil groups. 

All of the animal studies were also approved by the Ethics Committee of Tehran University of Medical Sciences and experiments were performed in compliance with the National Institutes of Health Guide for Care and Use of Laboratory Animals (publication No. 85-23, revised 2007). 

**Experimental design**

**Vaginal smear**

Estrous cyclicity was monitored by vaginal smears obtained between 0800 and 1200 hours, and it was assessed by analysis at the light microscopy level of the relative proportion of leukocytes, epithelial and cornified cells found in daily vaginal lavages, which characteristically change during different stages of the estrous cycle. The rat estrous cycle (estrus, diestrus1, diestrus2, and proestrus) usually lasts about 4 days, in controls or PCO rats (23).

**Hormonal treatment and study procedure**

After 1 week of acclimatization, smear is taken from 7-8 week-old rats (n=24) and after 4 days each rat receives an i.m. injection of Estradiol Valerate ( Aburaihan Co., Iran), 2mg in 0.2 ml of seasame oil, to induce PCO as described by Brawer 1996 (24). 

Control rats (n=6) were injected with sesame oil. “All experiments were performed 60 days after the injection, when follicular cysts are first detected”. Then PCO rats and sesame oil groups were subdivided in two groups: LC lesion and without LC lesion (normal LC) groups. The dose of yohimbine (Yoh) in this study was 0.4mg/kg according to dose response trial in two groups: Se-Yoh and Est-Yoh. Vaginal smears were taken daily to verify estrous cycle regularity, food and water were provided ad libitum. 

**Study groups**

(A) LC lesion

A1→Injection of estradiol valerate for PCO modeling (*n*=6)

A2→Injection of sesame oil control for PCO modeling (*n*=6)

(B) Control (without LC lesion)

B1→Injection of estradiol valerate for PCO modeling (*n*=6)

B2→Injection of sesame oil control for PCO modeling (*n*=6)

(C) Yohimbine

C1→Injection of estradiol valerate for PCO modeling (*n*=6)

C2→Injection of sesame oil control for PCO modeling (*n*=6)

(D) Intact group

**LC neurochemical lesion**

Under ketamine 100 mg/kg body weight, ip and xylazine 14 mg/kg body weight, ip anesthesia, rats were positioned in a stereotaxic instrument with the incisor bar set at the zero point. The dorsal surface of the skull was exposed, and holes, 2 mm in diameter, were drilled bilaterally. Bilateral LC lesions were made using an SEG 5 gl syringe. 

The coordinates were: AP=-0.8 mm (interaural), L=+0.9ram (bregma) and V=+3.0ram (interaural), (Paxinos and Watson, 1986) under an angle of 30 o in rostro-caudal direction. Each side was injected with 2μl 6-OHDA hydrobromide (Sigma-Aldrich Chemie GmbH, Deisenhofen, Germany) (6μg/μl, dissolved in 0.1mg/ml ascorbic acid; injection time: 0.4μl/min) (25).

**Measurement of circulating levels of gonadotropins and gonadal steroid**

Blood samples were collected from the heart and centrifuged at 3500*g *for 10 min. Serum was isolated and immediately frozen at -80°C. Serum luteinising hormone (LH), follicular stimulation hormone (FSH) and esteradiol levels were determined by ELISA. 

“Kits were used to measure LH (Kit CA-92627 Cod. No. EIA-4K2G5, from American Co. Monobind, Inc. Costa Mesa), FSH (Kit CA-92627 Cod.No. EIA-6K2G5 from American Co. Monobind, Inc. Costa Mesa) and Esteradiol (Kit DRG Cod. No. EIA-2693 from American Co. DRG International GmbH)”.

**Ovarian morphology**

The ovaries from controls (EV-treated), LC lesion and yohimbine treated were removed, cleaned of adherent connective fat tissue, and fixed in 10% formaldehyde buffer for at least 24 hours. Ovaries were imbedded in paraffin, cut in 8-µm sections, and stained with hematoxylin and eosin.

**Statistical analysis**

Data are presented as mean±SEM. Statistical differences were determined by two-way and one-way ANOVA followed by the Bonferroni post hoc test. SPSS-13 was used for data analysis. p<0.05 was considered as significant level.

## Results

**LC lesion and yohimbine administration effects on plasma levels of ovarian steroids and gonadotropins**

**Figure 1:**
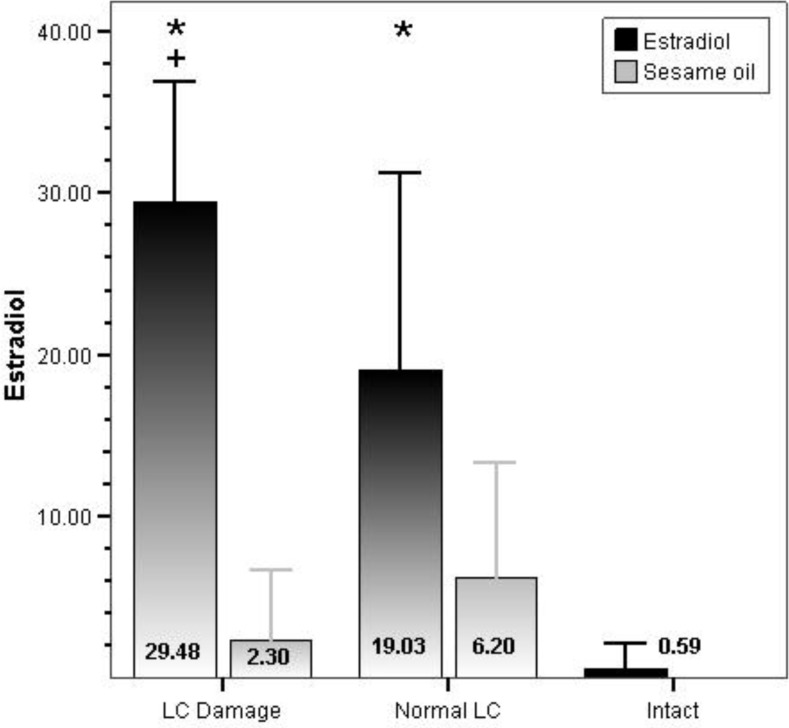
Comparison of serum level of estradiol between LC Lesion groups and control

The serum level of Estradiol was significantly increased in PCO rat with (p<0.001) and without (p<0.001) LC lesion in comparison with intact group. Also there was a significant difference between serum level of Estradiol in PCO and non PCO rat with LC lesion (p=0.001) (Figure 1). 

**Figure 2:**
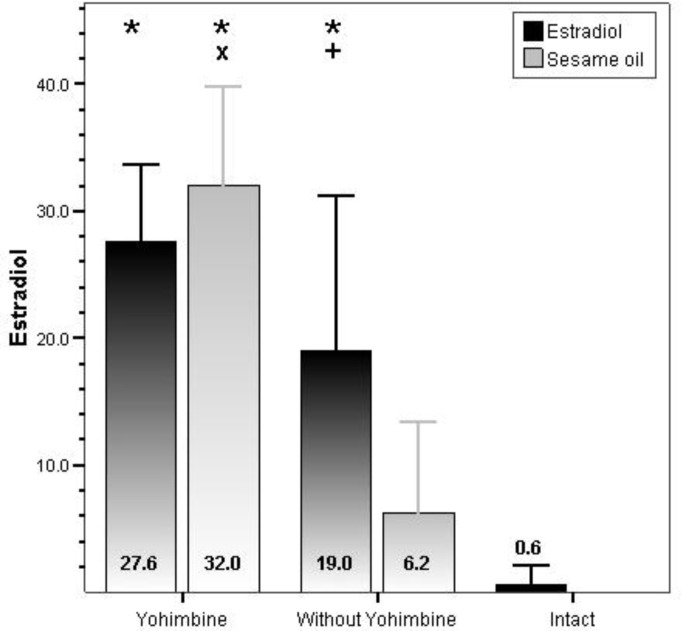
Comparison of serum level of estradiol between yohimbine administration and control group.

In yohimbine groups estradiol concentration were significantly increased by systemic administration in comparison with control (p<0.001) and intact group (p<0.001) (Figure 2). No significant changes were noted in the levels of FSH and LH in all groups (Table I and II). In PCO and control (sesame oil) groups of yohimbine there was significantly increasing of estradiol level and corpora lutea.

**Histological findings**

**LC lesion and yohimbine administration upon ovarian morphology**

**Figure 5:**
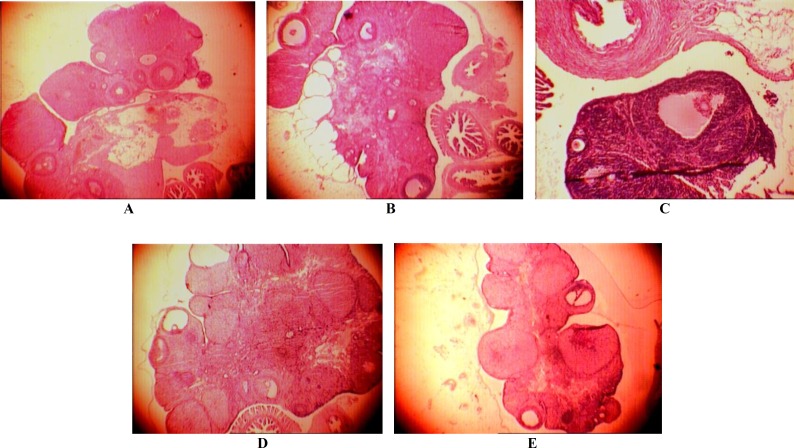
LC lesion and yohimbine effects upon ovarian morphology: A, Ovarian morphology on estrus of rat maintained at ambient temperature (intact /control); B, Ovarian morphology on EV treated rats (PCO). Ovarian morphology on LC lesion on PCO rats D, is ovarian morphology on yohimbine effect in PCO rats. E, Ovarian morphology of sesame oil rat as control of EV with yohimbine

The morphological analyses of ovaries from control rats on estrus revealed the presence of numerous corpora lutea in different stages of development and regression, many of which clearly resulted from recent ovulation, as well as some atretic antral follicles (Figure 5A). 

Ovarian morphology on estrus of rats exposed to Estradiol Valerate (Figure 5B) and LC lesion induced several ovarian morphological alterations, marked predominantly by the presence of numerous healthy antral follicles with small size and follicles with enlarged theca cell layer (hyperthecosis) (Figure 5C) and cyst. 

Ovulation with corpora lutea formation occurred during yohimbine administration together with decreasing of cysts number (Figure 5D).

**Table I:** Comparison of hormonal level between PCO and non PCO rat with LC normal and lesion

**Study group**		**n**	**Serum level of**		
**PCO **	** LC **		**Estradiol ** ***)*** ***Pg/ml)***	**FSH ** ***(IU/ml)***	**LH ** ***(IU/ml)***
Estradiol Valerate	lesion	4	29.48±7.45^A^^B^	0.000±0.000	0.675±0.822
Sesame oil (control)	lesion	6	2.30±4.36	0.167±0.197	0.450±0.686
Estradiol Valerate	normal	7	19.03±12.18^A^	0.271±0.281	5.714±7.677
Sesame oil (control)	normal	7	6.20±7.19	0.286±0.146	4.829±8.481
Intact		8	0.59±1.55	0.200±0.334	1.525±3.500

A : Significant difference in comparison with intact group.

B : Significant difference in comparison between PCO and non PCO groups.

**Table II:** Comparison of hormonal level between PCO and non PCO rat with and without yohimbine

**Study group**		**n**	**Serum level of**		
**PCO **	**Yohimbine **		**Estradiol ** ***)*** ***Pg/ml)***	**FSH ** ***(IU/ml)***	**LH ** ***(IU/ml)***
Estradiol Valerate	with	6	27.55±6.12^A^	0.050±0.084	1.167±1.778
Sesame oil(control)	with	6	32.02±7.76^A^^C^	0.083±0.098	2.983±2.555
Estradiol Valerate	without	8	19.03±12.18^A^^B^	0.238±0.277	6.263±7.275
Sesame oil(control)	without	7	6.20±7.19	0.286±0.146	4.829±8.481
Intact		7	0.59±1.55	0.229±0.350	0.300±0.532

A : Significant difference in comparison with intact group.

B : Significant difference in comparison between PCO and non PCO groups.

C : Significant difference in comparison between groups with and without yohimbine.

*: Significant difference in comparison with intact group.

+: Significant difference in comparison between PCO and non PCO groups.

*: Significant difference in comparison with intact group.

+: Significant difference in comparison between PCO and non PCO groups.

×: Significant difference in comparison between groups with and without yohimbine.

## Discussion

The aim of this study was to test the hypothesis that sympathetic nervous system has a critical role in creation of poly cystic ovary syndrome modeling in rat. The noradrenergic nucleus locus coeruleus (LC) has been reported to regulate LHs secretion in female rats. 

Oestrogen receptors have been demonstrated in LC neurones, suggesting that these cells are possibly responsive to variations in circulating levels of ovarian steroids. Oestradiol has been reported to regulate the gene expression of noradrenaline biosynthetic enzymes in the LC (26), “but the effects of ovarian steroids on the activity of LC neurones and the relevance of such actions for LH secretion remain poorly understood”. 

The locus coeruleus (LC) is a densely packed cell group located in the dorsal pons which contains approximately half of all noradrenergic neurons in the rat brain. Females have a higher number of LC neurons than males, and this difference appears to depend on estradiol receptor- β (ER-β) (27). 

The neurons arising in the LC, project directly to the GnRH-rich regions of the preoptic area (28) and selective lesioning of the LC results in a decrease in noradrenaline levels within the preoptic area (29), hypothalamus and prevents the occurrence of the LH surge (30). LC noradrenergic neurons may directly alter the activity of the GnRH neural system. Numerous studies implicated noradrenaline as an important regulator of GnRH/LH release. 

Infusion of noradrenaline either intracerebroventricularly or directly into the preoptic area of the rat suppresses LH pulses (31). It is interesting to note that near complete interruption of ascending noradrenergic pathways did not alter LH pulse frequency (32), whereas discrete lesions of the LC, destroying 50% or more of the nucleus, resulted in a permanent inhibition of pulsatile LH release (33). 

Recently, it was demonstrated that women with PCOS have enhanced ovarian productions of nerve growth factor (NGF), a strong marker of sympathetic nerve activity. These results suggest that overproduction of ovarian NGF is a component of PCO morphology in humans. In a transgenic mouse model over expressing NGF in the ovaries, persistent elevation in plasma LH levels is required for the morphological abnormalities to appear (34). The transection of the superior ovarian nerve in the EV-induced rat PCO model reduces the steroid response, increases β_2_-adrenoceptor expression to more normal levels, and restores estrus cyclicity and ovulation (35). 

Also, blockade of endogenous NGF action restores the EV-induced changes in ovarian morphology and expression of the sympathetic markers α_1_- and β_2_-adrenoceptors, p75 neurotrophin receptor p^75NTR^, NGF-tyrosine kinase receptor, and tyrosine hydroxylase. These data confirm the close interaction between NGF and the sympathetic nervous system in the pathogenesis of steroid-induced PCO in rats (36). 

In contrast, however, peripheral administration of -adrenergic receptor antagonists also decreases the frequency of the GnRH pulse generator (37). Our data from this study suggest that, LC noradrenergic neurons have a critical role in feedback system of estradiol because its lesion in PCO rats increased estradiol level and induced hyperthecosis (38), that shows LC lesion cause latency in the processing of modeling PCO in rat. 

Yohimbine as the α2 receptor antagonist showed increasing estradiol level (like LC lesion) and numbers of corpus lutea and developed follicles for characterizing of the peripheral sympathetic nervous system in PCO rats. It is interesting to note that data of yohimbine shows that in sesame oil group there are increasing of estradiol level and corpus lutea that resembles PCO which seems to be the direct effect of this antagonist in rat ovary. 

We did not find changes in plasma LH and FSH levels in either groups of LC and yohimbine. Our results indicate that LC lesion and yohimbine have effectiveness in processing of modeling of PCOS in rat. These findings support the theory that increased sympathetic activity contributes to the development and maintenance of PCOS.

## References

[1] Azziz R, Woods KS, Reyna R, Key TJ, Knochenhauer ES, Yildiz BO (2004). The prevalence and features of the polycystic ovary syndrome in an unselected population. J Clin Endocrinol Metab.

[2] Norman RJ, Dewailly D, Legro RS, Hickey TE (2007). Polycystic ovary syndrome. Lancet.

[3] Aguado LI (2002). Role of the central and peripheral nervous system in the ovarian function. Microsc Res Tech.

[4] Honma K, Wuttke W (1980). Norepinephrine and dopamine turnover rates in the medial preoptic area and the mediobasal hypothalamus of the rat brain after various endocrinological manipulations. Endocrin.

[5] Rothfeld J, Hejtmancik JF, Conn PM, Pfaff DW (1989). In situ hybridization for LHRH m RNA following estrogen treatment. Mol Brain Res.

[6] Simonian SX, Delaleu B, CaratyA, HerbisonAE (1998). Estrogen receptor expression in brainstem noradrenergic neurons of the sheep. Neuroendocrin.

[7] Swanson LW, Hartman BK (1976). The central adrenergic system. A immunofluorescence study of the location of cell bodies and their efferent connections in the rat using dopamine-b-hydroxylase as a marker. J Comp Neurol.

[8] Wise PM, Rance N, Selmanoff M, Barraclough CA (1981). Changes in radio immune assayable luteinizing hormone-releasing hormone in discrete brain areas of the rat at various times on proestrus, diestrous day 1 and after phenobarbital administration. Endocrin.

[9] Stener-Victorin E, Jedel E, Manneras L (2008). Acupuncture in Polycystic Ovary Syndrome: Current Experimental and Clinical Evidence. J Neuroendocrinol.

[10] Fagius J (2003). Sympathetic nerve activity in metabolic control-some basic concepts. Acta Physiol Scand.

[11] Hoffman LK, Ehrmann DA (2008). Cardiometabolic features of polycystic ovary syndrome. Nat Clin Pract Endocrinol Metab.

[12] Solomon CG (1999). The epidemiology of polycystic ovary syndrome Prevalence and associated disease risks.. Endocrinol Metab Clin North Am.

[13] Nelson VL, Qin KN, Rosenfield RL, Wood JR, Penning TM, Legro RS (2001). The biochemical basis for increased testosterone production in theca cells propagated from patients with polycystic ovary syndrome. J Clin Endocrinol Metab.

[14] Sverrisdóttir YB, Mogren T, Kataoka J, Janson PO, Stener-Victorin E (2008). Is polycystic ovary syndrome associated with high sympathetic nerve activity and size at birth?. Am J Physiol Endocrinol Metab.

[15] Berridge CW, Waterhouse BD (2003). The locus coeruleus-noradrenergic system: modulation of behavioral state and state-dependent cognitive processess. Brain Res Rev.

[16] Kvetnansky R, Bodnar I, Shahar T, Uhereczky G, Krizanova O, Mravec B (2006). Effect of lesion of A5 and A7 brainstem noradrenergic areas or transaction of brainstem pathways on sympathoadrenal activity in rats during immobilization stress. Neurochem Res.

[17] Sved AF, Cano G, Card JP (2001). Neuroanatomical specificity of the circuits controlling sympathetic outflow to different targets. Clin Exp Pharmacol.

[18] Bernuci MP, Szawka RE, Helena CV, Leite CM, Lara HE, Anselmo-Franci JA (2008). Anselmo-Franci. Locus Coeruleus Mediates Cold Stress-Induced. Polycystic Ovary in Rats. Endocrin.

[19] Anselmo-Franci JA, Franci CR, Krulich L, Antunes-Rodrigues J, McCann SM (1997). Locus coeruleus lesions decrease norepinephrine input into the medial preoptic area and medial basal hypothalamus and block the LH, FSH and prolactin preovulatory surge. Brain Res.

[20] Vega Helena CV, Franci CR, Anselmo-Franci JA (2002). Luteinizing hormone and luteinizing hormone-releasing hormone secretion is under locus coeruleus control in female rats. Brain Res.

[21] Barria A, Leyton V, Ojeda SR, Lara HE (1993). Ovarian steroidal response to gonadotropins and beta-adrenergic stimulation is enhanced in polycystic ovary syndrome: role of sympathetic innervation. Endocrinology.

[22] Lara HE, Ferruz JL, Luza S, Bustamante DA, Borges Y, Ojeda SR (1993). Activation of ovarian sympathetic nerves in polycystic ovary syndrome. Endocrinology.

[23] Szukiewicz D, Uilenbro M (1998). Polycystic ovary syndrome-searching for an animal model. J Med.

[24] Brawer JR, Munoz M (1996). Development of the polycystic ovarian conditions (PCO) in the estradiol valerate-treated rat. Biol Reprod.

[25] Riickert N, Bubser M, Schmidt WJ (1997). 6-Hydroxydopamine lesion of locus coeruleus and the antiparkinsonian potential of NMDA-receptor antagonists in rats. J Neural Transm.

[26] Serova L, Rivkin M, Nakashima A, Sabban EL (2002). Estradiol stimulates gene expression of norepinephrine biosynthetic enzymes in rat locus coeruleus. Neuroendocrinology.

[27] Pendergast JS, Tuesta LM, Bethea JR (2008). Oestrogen receptor beta contributes to the transient sex difference in tyrosine hydroxylase expression in the mouse locus coeruleus. J Neuroendocrinol.

[28] Wright DE, Jennes L (1993). Origin of noradrenergic projections to GnRH perikarya-containing areas in the medial septum-diagonal band and preoptic area. Brain Res.

[29] Anselmo-Franci JA, Franci CR, Krulich L, Antunes-Rodrigues J, McCann SM (1997). Locus coeruleus lesions decrease norepinephrine input into the medial preoptic area and medial basal hypothalamus and block the LH, FSH and prolactin preovulatory surge. Brain Res.

[30] Helena CV, Franci CR, Anselmo-Franci JA (2002). Luteinizing hormone and luteinizing hormone-releasing hormone secretion is under locus coeruleus control in female rats. Brain Res.

[31] Gallo RV, Drouva SV (1979). Effect of intraventricular infusion of catecholamines on luteinizing hormone release in ovariectomized and ovariectomized, steroid-primed rats. Neuroendocrinology.

[32] Leipheimer RE, Gallo RV (1985). Medial preoptic area involvement in norepinephrine-induced suppression of pulsatile luteinizing hormone release in ovariectomized rats. Neuroendocrinology.

[33] Clifton DK, Steiner RA (1985). Recovery of pulsatile luteinizing hormone secretion following permanent disruption of the ascending noradrenergic fiber tract in the ovariectomized rat. Biol Reprod.

[34] Anselmo-Franci JA, Rocha-Barros VM, Franci CR, McCann SM (1999). Locus ceruleus lesions block pulsatile LH release in ovariectomized rats. Brain Res.

[35] Dissen GA, Garcia-Rudaz C, Ojeda SR (2009). Role of neurotrophic factors in early ovarian development. Semin Reprod Med.

[36] Barria A, Leyton V, Ojeda SR, Lara HE (1993). Ovarian steroidal response to gonadotropins and beta-adrenergic stimulation is enhanced in polycystic ovary syndrome: role of sympathetic innervation. Endocrinology.

[37] Manni L, Lundeberg T, Holmang A, Aloe L, Stener-Victorin E (2005). Effect of electro-acupuncture on ovarian expression of alpha (1)- and beta (2)-adrenoceptors, and p75 neurotrophin receptors in rats with steroid-induced polycystic ovaries. Reprod Biol Endocrinol.

[38] Kaufman JM, Kesner JS, Wilson RC, Knobil E (1985). Electrophysiological manifestation of luteinizing hormone-releasing hormone pulse generator activity in the rhesus monkey: influence of -adrenergic and dopaminergic blocking agents. Endocrinology.

[39] Bernuci MP, Szawka RE, Helena CVV, Leite CM, Lara HE, Anselmo-Franci JA (2008). Locus Coeruleus Mediates Cold Stress-Induced Polycystic Ovary in Rats. Endocrinology.

